# The Kiwifruit Emerging Pathogen *Pseudomonas syringae* pv. *actinidiae* Does Not Produce AHLs but Possesses Three LuxR Solos

**DOI:** 10.1371/journal.pone.0087862

**Published:** 2014-01-31

**Authors:** Hitendra Kumar Patel, Patrizia Ferrante, Sonia Covaceuszach, Doriano Lamba, Marco Scortichini, Vittorio Venturi

**Affiliations:** 1 International Centre for Genetic Engineering and Biotechnology, Trieste, Italy; 2 Research Centre for Fruit Crops, Agricultural Research Council, Roma, Italy; 3 Research Unit for Fruit Trees, Agricultural Research Council, Caserta, Italy; 4 Istituto di Cristallografia, Consiglio Nazionale delle Ricerche, U.O.S di Trieste, Trieste, Italy; The Scripps Research Institute and Sorrento Therapeutics, Inc., United States of America

## Abstract

*Pseudomonas syringae* pv. *actinidiae* (*Psa*) is an emerging phytopathogen causing bacterial canker disease in kiwifruit plants worldwide. Quorum sensing (QS) gene regulation plays important roles in many different bacterial plant pathogens. In this study we analyzed the presence and possible role of *N*-acyl homoserine lactone (AHL) quorum sensing in *Psa*. It was established that *Psa* does not produce AHLs and that a typical complete LuxI/R QS system is absent in *Psa* strains. *Psa* however possesses three putative *luxR* solos designated here as PsaR1, PsaR2 and PsaR3. PsaR2 belongs to the sub-family of LuxR solos present in many plant associated bacteria (PAB) that binds and responds to yet unknown plant signal molecules. PsaR1 and PsaR3 are highly similar to LuxRs which bind AHLs and are part of the canonical LuxI/R AHL QS systems. Mutation in all the three *luxR* solos of *Psa* showed reduction of *in planta* survival and also showed additive effect if more than one solo was inactivated in double mutants. Gene promoter analysis revealed that the three solos are not auto-regulated and investigated their possible role in several bacterial phenotypes.

## Introduction

Quorum sensing (QS) is an intercellular communication system in bacteria that links bacterial cell density to gene expression via the production and detection of signal molecules [1], [2]. In Gram-negative bacteria, *N*-acyl homoserine lactones (AHL) signal molecules are most commonly used; they are produced by an AHL synthase, which belongs to the LuxI-protein family and a transcriptional regulator belonging to the LuxR family. The LuxR-family protein forms a complex with the cognate AHL at threshold (‘quorum’) concentration and affects the transcription of target genes [3]. QS-dependent regulation in bacteria is most often involved in the coordinated community action of bacteria like antibiotic production, biofilm formation, conjugation, bioluminescence, production of extracellular enzymes, virulence factors and pigment formation [2], [4]–[6]. Well-characterized examples of QS-dependent regulation of phenotypic functions in *Pseudomonas* include the LasI/LasR and RhlI/RhlR of the opportunistic human pathogen *P. aeruginosa* [7], [8], the AhlI/AhlR system of the plant pathogen *P. syringae* pv. *syringae* [9], the PfsI/PfsR and PfvI/PfvR of the emerging plant pathogen *P. fuscovaginae* [10], the PcoI/R system of *P. corrugata* [11], the two QS systems PhzI/R and CsaI/R of plant beneficial *P. aureofaciens* [12]–[14], the PupI/PupR of plant growth-promoting *P. putida* [15], [16], and the MupI/MupR QS system of plant growth promoting *P. fluorescencs* NCIMB 10586 [17].

The AHLs molecules produced by different LuxI-family synthases vary in length of the acyl chain (from 4 to 18 carbon atoms) and in their substitution (eg an hydroxyl or oxo substitution) in the third carbon position of the acyl chain [3]. LuxR proteins are approximately 250 amino acids long and consist of two domains; an AHL-binding domain at the *N*-terminal region [18], [19] and a DNA-binding helix-turn-helix (HTH) domain at the C-terminal region [20]–[22]. The AHL-binding domain recognizes AHLs most often resulting in its ability to bind target DNA in gene promoter regions at a conserved sites called a *lux* box [23], [24]. QS LuxRs display surprisingly low homologies (18–25%); 95% however share 9 highly conserved amino acid residues [6], [25]. Six of these are hydrophobic or aromatic and form the cavity of the AHL-binding domain and the remaining three are in the HTH domain [26].

In a typical AHL QS system, *luxI/R* genes are almost always located genetically adjacent to each other. In many proteobacteria, additional QS *luxR*-type genes also have been found that are unpaired to a cognate *luxI* synthase. An analysis of 265 proteobacterial genomes by Case *et al*. in 2008 showed that 68 had a canonical paired *luxI/R* system and out of these 68, 45 contained more *luxRs* than *luxIs*; another set of 45 genomes contained only QS *luxR* genes. These QS LuxR proteins lacking a genetically linked LuxI have been termed “orphans” [27] and more recently “solos” [28]. LuxR solos have the same modular structure; an AHL binding domain in the *N*-terminus and a DNA binding HTH domain at the C-terminus as like other LuxRs from canonical LuxI/R systems. LuxR solos can result in the increase of the regulatory targets of the canonical complete AHL QS systems by responding to endogenous AHLs or they are responsible for eavesdropping by detecting exogenous AHLs molecules. For example, QscR from *P. aeruginosa* responds to endogenously produced AHLs [29], [30] while SdiA of *Salmonella enterica* and *E. coli* which do not produces AHLs and eavesdrops on AHLs produced by neighboring bacteria [31]–[34].

A sub-group of LuxR solos has been recently discovered which are only found in plant-associated bacteria (PAB) that do not bind AHLs but to plant produced compounds [35], [36]. These LuxRs are very closely associated to QS LuxRs differing in the conservation of one or two of the six highly conserved amino acids in the AHL-binding domain. Five members of this subfamily have been studied and these are XccR of *Xanthomonas campestris* pv. *campestris* (*Xcc*), OryR of *Xanthomonas oryzae* pv. *oryzae* (*Xoo*), PsoR of *Pseduomonas fluorescens*, XagR of *Xanthomonas axonopodis* pv. *glycines* (*Xag*) and NesR in *Sinorhizobium meliloti* [37]–[42]. OryR of the rice vascular pathogen *Xoo* is involved in virulence, it responds to plant signals and activates the expression of the neighboring proline iminopeptidase (*pip*) and of motility genes [38], [39], [43]. XccR of the crucifer pathogen *Xcc* also responds to a yet unidentified plant compound and regulates the neighboring *pip* gene [42]. XagR of the soybean pathogen *Xag* which causes bacterial leaf pustule on soybean (*Glycine max*) is also involved in virulence [37]. Like XccR in *Xcc* and OryR in *Xoo*, also XagR in *Xag* activates *pip* transcription *in planta* thought to be due to a plant compound(s) which are over-produced by the plant in response to pathogen attack by *Xag*. Two of these PAB LuxR-type proteins have been studied in plant-beneficial bacteria, namely PsoR of *P. fluorescens* and NesR of *S. meliloti* [40], [41]. PsoR responds to plant compounds of several plant species and plays a role in biocontrol in rhizospheric *P*. *fluorescens* [41], NesR of *S. meliloti* has been associated with survival under stress and utilization of various carbon sources [40].

*Pseudomonas syringae* pv. *actinidiae* (*Psa*) is one of the emerging pathogens of *Pseudomonas* group which causes trunk canker, twig wilting and leaf spot on kiwifruit species (*Actinidia deliciosa* and *chinensis*) [44]. *Psa* was first described in Japan in 1984 [45] and later was isolated in South Korea [46] and Italy [47]. In 2008, a re-emergence of *Psa* was found on *A. chinensis* (kiwigold) plants in central Italy and caused a huge economic loss [48]. This outbreak was caused by a different population of *Psa* from the original one that caused fewer problems in 1992 [49]. More recently, *Psa* has been isolated in several countries including China [50], Portugal [51], France [52], Australia [53], Chile [54], New Zealand [55], Spain [56], Switzerland [57] and Turky [58].

The virulence mechanisms of *Psa* are largely unknown since no significant genetic and molecular studies have been thus far performed. Comparative genomic studies comparing genome sequences of several *Psa* strains suggests that a canonical AHL QS system is absent in *Psa* [49], [59]–[61]. Here we report that *Psa* does not produce AHLs and does not contain a complete canonical AHL QS system but possesses three LuxR solos. One of these belongs to the sub-family of PAB solos which do not respond to AHLs but to plant signals [41]. The other two LuxR solos might be involved in the signaling with neighboring bacteria by AHLs eavesdropping. Here we also report genetic studies of these three solos and their potential roles in virulence.

## Results and Discussion

### A Canonical AHL QS System is Absent in *P. syringae* pv. *actinidiae* (*Psa*)

It was of interest to determine if *Psa* produces AHLs and thus possesses a canonical AHL QS system. Purification of AHLs was performed on spent supernatants of 11 *Psa* isolates of Italy both from kiwigreen (*A. deliciosa*) and kiwigold (*A. chinensis*) (see Table 1); it was established that all the *Psa* strains did not produce detectable AHL molecules in TLC plates using three different AHL bacterial biosensors which can detect a wide range of structurally different AHLs (see Materials and Methods and data not shown). These results suggest that AHL mediated LuxI/R type QS is either absent in *Psa* strains or AHLs could not be detected by the analysis performed in this study as these might be produced in very low quantities or have structures which are not detected by the sensors. Soon after this analysis, the draft genome sequences of several *Psa* strains from Italy [49] and later from China, New Zealand, Japan, Korea and Chile were published [59]–[61]; none of these genomes contained canonical *luxI/R* pairs. It was concluded that *Psa* does not produce AHLs and consequently does not possess a complete AHL QS system. Plant pathogenic *P. syringa*e strains display diverse and host-specific interactions with different plant species. Specific strains are classified to one of the over 50 known pathovars based on their ability to infect various plant species (www.pseudomonas-syringae.org). A few pathovars have been reported to be able to produce AHLs, these include *P. syringae* pv. *syringae, P. syringae* pv. *tabaci, P. syringae* pv. *maculicola* [9], [62], [63]; however many are believed not to produce AHL signal molecules.

**Table 1:** Bacterial strains used in this study.

Strains	Relevant characteristics^a^	Reference/Source
***E. coli***		
DH_5_a	Cloning strain, Nal^r^	[72]
PRK2013	Helper strain for tri-parental conjugation, Km^r^	[77]
*E. coli* (pSB401)	Biosensor strain; Tc^r^	[70], [73]
***Biosensors***		
*A. tumefaciens* NT1	Harbouring pZLR4 plasmid, β-galactosidase reporter system, Gm^r^	[95]
*C. violaceum* CV026	Violacin pigment reporter system, Km^r^	[79]
***Pseudomonas syringae*** ** pv. ** ***actinidiae*** ** (** ***Psa*** **)**		
*Psa* 10,22	Wild type; Italian isolate; Nf^r^	Lab collection
*Psa* 10,24	Wild type; Italian isolate; Nf^r^	Lab collection
*Psa* 10,25	Wild type; Italian isolate; Nf^r^	Lab collection
*Psa* 10,29	Wild type; Italian isolate; Nf^r^	Lab collection
*Psa* 10,30	Wild type; Italian isolate; Nf^r^	Lab collection
*Psa* 11,41	Wild type; Italian isolate; Nf^r^	Lab collection
*Psa* 11,47	Wild type; Italian isolate; Nf^r^	Lab collection
*Psa* 11,50	Wild type; Italian isolate; Nf^r^	Lab collection
*Psa* 11,51	Wild type; Italian isolate; Nf^r^	Lab collection
*Psa* 12,56	Wild type; Italian isolate; Nf^r^	Lab collection
*Psa* 12,64	Wild type; Italian isolate; Nf^r^	Lab collection
*Psa*-mR1	*psaR1*::pKNOCK; Nf^r^, Km^r^; derivative of wild type	This work
*Psa*-mR2	*psaR2*::pKNOCK; Nf^r^, Km^r^; derivative of wild type	This work
*Psa*-mR3	*psaR3*:: in-frame deletion mutant generated by pEX19Gm plasmid; Nf^r^;derivative of wild type	This work
*Psa*-mR1+pBBR-*psaR1*	*Psa*-mR1 carrying full length *psaR1* in pBBR; Nf^r^, Km^r^, Gm^r^; derivative of *Psa*-mR1	This work
*Psa*-mR2+pBBR-*psaR2*	*Psa*-mR2 carrying full length *psaR2* in pBBR; Nf^r^, Km^r^, Gm^r^; derivative of *Psa*-mR2	This work
*Psa*-mR3+pBBR-*psaR3*	*Psa*-mR3 carrying full length *psaR3* in pBBR; Nf^r^, Gm^r^, derivative of *Psa*-mR3	This work
*Psa*-mR3+pcos-*psaR3*	*Psa*-mR3 carrying cosmid clone for *psaR3*; Nf^r^, Tc^r^, derivative of *Psa*-mR3	This work
*Psa*-mR3+ *Psa*-mR1	*Psa*-mR3 and *Psa*-mR1 double mutant; Nf^r^, Km^r^; derivative of *Psa*-mR3	This work
*Psa*-mR3+ *Psa*-mR2	*Psa*-mR3 and *Psa*-mR2 double mutant; Nf^r^, Km^r^; derivative of *Psa*-mR3	This work
*Psa*-mR3+ *Psa*-mR1+ pcos-*psaR3*+pBBR-*psaR1*	*Psa*-mR3 and *Psa*-mR1 double mutant carrying cosmid clone for *psaR3* and fulllength *psaR1* in pBBR; Nf^r^, Km^r^, Tc^r^, Gm^r^; derivative of *Psa*-mR3+ *Psa*-mR1	This work
*Psa*-mR3+ *Psa*-mR2+ pcos-*psaR3*+pBBR-*psaR2*	*Psa*-mR3 and *Psa*-mR2 double mutant carrying cosmid clone for *psaR3* and fulllength *psaR2* in pBBR; Nf^r^, Km^r^, Tc^r^, Gm^r^; derivative of *Psa*-mR3+ *Psa*-mR2	This work

a Nal^r^, Km^r^, Tc^r^, Gm^r^, and Nf^r^ indicate resistance to nalidixic acid, kanamycin, tetracycline, gentamycin and nitrofurantoin respectively.

### *Psa* has Two QS LuxR Solos and One PAB LuxR Solo

Numerous sequenced proteobacterial genomes have QS-related LuxR AHL sensors/regulators which lack a cognate LuxI AHL synthase [64]. These unpaired QS LuxR-family proteins have been recently called solos [28] and possess the typical modular structure having an acyl-homoserine lactone binding domain at their *N*-terminus and a helix-turn-helix DNA binding domain at their *C*-terminus. Interestingly *Psa* possesses three such LuxR solos (Table S3), designated here as PsaR1, PsaR2 and PsaR3, and these could be playing roles in detecting and responding to exogenous signals. One of these solos, PsaR2, most likely belongs to a sub-family of LuxR solos only found in plant-associated bacteria (PAB) which binds and responds to yet unknown plant signals [35]. This family has an imperfect AHL-binding domain with substitutions either in one or two of the highly conserved amino acids in the AHL binding domain, more precisely, W57 and Y61 (numbers in respect to TraR) were found substituted by methionine (M) and tryptophan (W) respectively (see below; Table 2). The other two LuxR solos appear to be related to AHL-LuxRs as they shared the nine conserved amino acid residues that were shown to be important for AHL binding (Table 2) [6], [25]. Generation of a phylogenetic tree for the subset of LuxR proteins that we used for alignment indicated that PsaR2 was grouped with PAB LuxR solos from other plant associated *Pseudomonas* species. PsaR1 and PsaR3 were grouped with QS associated LuxR proteins further suggesting that these two LuxR solos might be binding to AHL signal molecules (data not shown). The neighboring genes of the three *luxR* solos were also mapped (as depicted in Table S3). Importantly, we found that *psaR*2 has adjacently located the *pip* gene encoding for an proline iminopeptidase; notably all the sub-family of PAB *luxR* solos genes possess this locus next to it [35].

**Table 2:** Nine key amino acids in the alignment of LuxR proteins from subset of plant associated bacteria.

Category of LuxRs	Subset of LuxR proteins from Plant associated bacteria	Key amino acids in auto-inducer binding domain						Key amino acids in HTH domain		
AHL LuxRs	AAZ50597.1-TraR-*At*	W57	Y61	D70	P71	W85	G113	E178	L182	G188
	AAA25874.1-LasR-*Pa*	W	Y	D	P	W	G	E	L	G
	AAC44036.1-RhlR-*Pa*	W	Y	D	P	W	G	E	L	G
	ACM50924.1-ExpR-*Pcc*	W	Y	D	P	W	G	E	L	G
	AAC38403.1-CarR-*Pcc*	W	Y	D	P	W	G	E	L	G
	AAA82097.1-EsaR-*Pantoea*	W	Y	D	P	W	G	E	L	G
PAB LuxR solos	YP_199907.1-OryR-*Xoo*	**M**	**W**	D	P	W	G	E	L	G
	ZP_08179515.1-LuxR-*Xcv*	**M**	**W**	D	P	W	G	E	L	G
	YP_242384.1-AhyR-*Xcc*	**M**	**W**	D	P	W	G	E	L	G
	NP_643297.1-AhyR-*Xac*	**M**	**W**	D	P	W	G	E	L	G
	XagR-*Xag*	**M**	**W**	D	P	W	G	E	L	G
	ZP_03522316.1-LuxR-*Re*	**M**	**W**	D	P	W	G	E	L	G
	YP_765467.1-LuxR-*Rl*	**M**	**W**	D	P	W	G	E	L	G
	AGG75406.1-NesR-*Sm*	**M**	**W**	D	P	W	G	E	L	G
	AAY94512.1-PsoR-*Pf*-5	W	**W**	D	P	W	G	E	L	G
	YP_004351870.1-NarL-*Pbb*	W	**W**	D	P	W	G	E	L	G
	YP_276356.1-LuxR-*Psp*	W	**W**	D	P	W	G	E	L	G
	BAD15091-LuxR-*Paf*	W	**W**	D	P	W	G	E	L	G
	ZP_07006441.1-AhyR-*Psn*	W	**W**	D	P	W	G	E	L	G
AHL LuxR solo	PsaR1-*Psa*	W	Y	D	P	W	G	E	L	G
PAB LuxR solo	PsaR2-*Psa*	W	**W**	D	P	W	G	E	L	G
AHL LuxR solo	PsaR3-*Psa*	W	Y	D	P	W	G	E	L	G

Position of nine key amino acids in LuxR proteins are indicated using AAZ50597.1-TraR-*At* as a reference sequence. Amino acid substitution in respective position has been indicated by bold letters. *At*; *Agrobacterium tumefaciens*, *Pa*; *Pseudomonas aeruginosa* PAO1, *Pcc*; *Pectobacterium carotovorum* subsp. *carotovorum*, *Pantoea*; *Pantoea stewartii* subsp. *stewartii* DC283, *Xoo*; *Xanthomonas oryzae* pv. *oryzae* KACC 10331, *Xcv*; *Xanthomonas vesicatoria* ATCC 35937, *Xcc*; *Xanthomonas campestris* pv. *campestris* str. 8004, *Xac*; *Xanthomonas axonopodis* pv. *citri* str. 306, *Xag*; *Xanthomonas axonopodis* pv. *glycines*, *Re*; *Rhizobium etli* GR56, *Rl*; *Rhizobium leguminosarum* bv. *viciae* 3841, *Sm*; *Sinorhizobium meliloti* 2011, *Pf*-5; *Pseudomonas protegens* Pf-5, *Pbb*; *Pseudomonas brassicacearum* subsp. *brassicacearum* NFM421, *Psp*; *Pseudomonas syringae* pv. *phaseolicola* 1448A, *Paf*; *Pseudomonas azotoformans*, *Psn*; *Pseudomonas savastanoi* pv. *savastanoi* NCPPB 3335, *Psa*; *Pseudomonas syringae* pv. *actinidiae*.

### *In-silico* 3D Architecture and Cartography of the Ligand Binding Sites in PsaR1, PsaR2 and PsaR3

**Figure 1:**
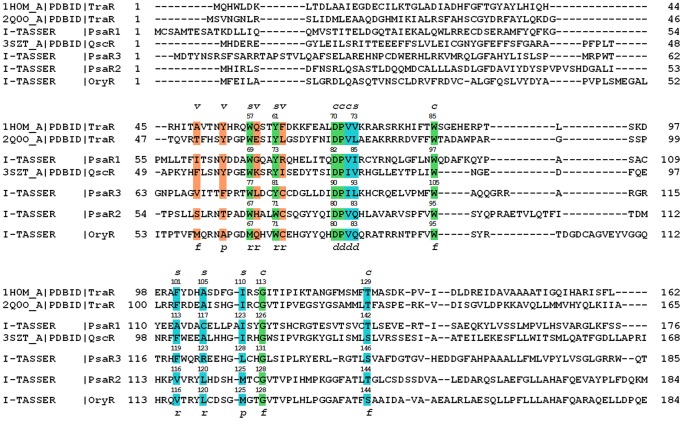
Structure-based multiple sequence alignment of the regulatory domains of the three *Psa* solos with QS LuxRs and with the prototype of the PAB LuxR solos subfamily. The residues belonging to **Cluster 1,** to **Cluster 2** and **Cluster 3** are highlighted in green, cyan and in orange, respectively. The 3D architecture of the boundaries of the ligand-binding site is schematized by ***r*** (roof), ***f*** (floor), ***p*** (proximal wall) and ***d*** (distal wall) and its tripartite topology by ***c*** (conserved core), ***s*** (specificity patch) and ***v*** (variable patch).

**Figure 2:**
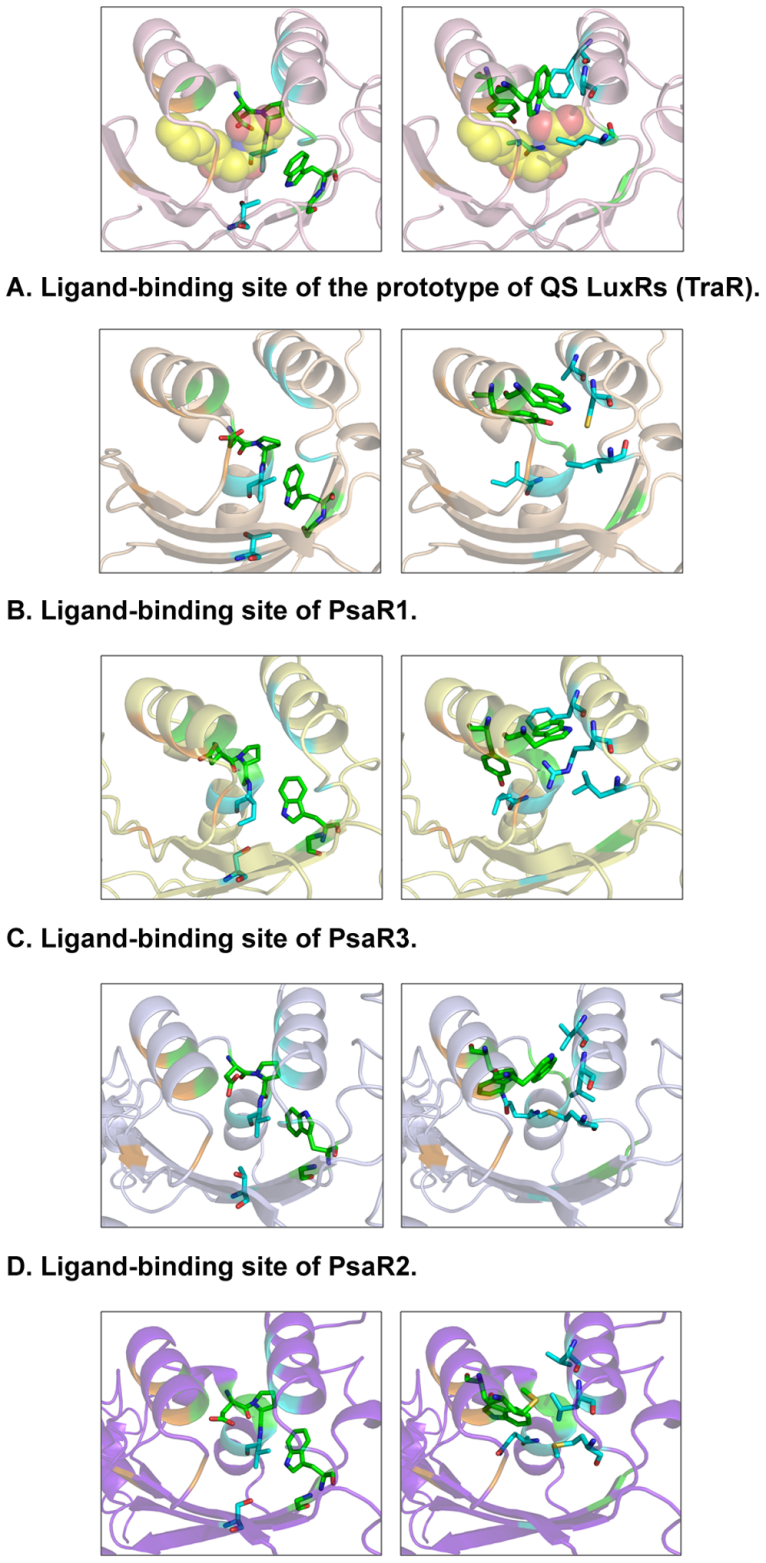
Comparison of the ligand-binding sites of the three *Psa* solos with the prototypes of QS LuxRs and PAB LuxR solos subfamily. Mapping the protein residues defining the three Clusters (**Cluster 1, Cluster 2** and **Cluster 3** colored in green, cyan and in orange, respectively) showing the amino acid side chains that delineate the conserved core (left column) and the specificity patch (right column), respectively on the X-ray crystal structure of TraR in complex with OC8-HSL (PDB_ID 1H0M) [68] (**A**) and on the 3D structure-based homology models of PsaR1 (**B**), PsaR3 (**C**), PsaR2 (**D**) and OryR (**E**). The carbon, nitrogen and oxygen atoms of the OC8-HSL ligand shown in (**A**) are represented by spheres and are colored in yellow, blue and red respectively. Figures produced by Pymol [94].

**Figure 3:**
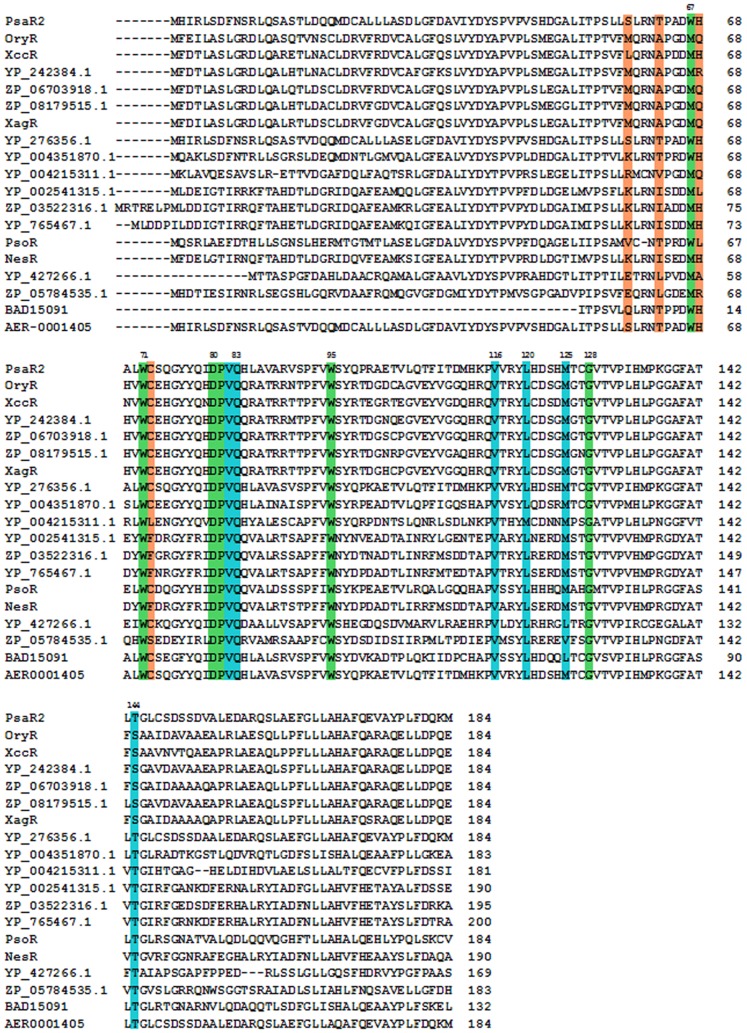
Multiple sequence alignment of the regulatory domain of PsaR2 with the PAB LuxR solos subfamily. The residues belonging to **Cluster 1, Cluster 2** and **Cluster 3** are highlighted in green, cyan and in orange, respectively.

In order to gain insights on PsaRs substrate specificity the 3D architecture and cartography of the ligand-binding sites have been dissected exploiting a structure-based homology model of the three LuxR solos obtained using I-TASSER [65]. The protein sequences of the PsaR1, PsaR2 and PsaR3 regulatory domains were structurally aligned to four different LuxR family proteins (Figure 1): three of them related to canonical LuxR family (TraR from *Sinorhizobium fredii* [PDB_ID 2Q0O] [66], QscR from *Pseudomonas aeruginosa* [PDB_ID 3SZT] [67] and TraR from *Agrobacterium tumefaciens* [PDB_ID 1H0M] [68] and one from PAB LuxR family (OryR from *Xanthomonas oryzae*). A comparative structural analysis of the cartography of the regulatory domains of PsaR1, PsaR2 and PsaR3 according to the 3D molecular descriptors [69] suggested that in addition to the six conserved hydrophobic/aromatic residues previously reported (Table 2) [6], [26] to delineate the binding site (named Cluster 1 and colored in green in Figures 1, 2 and 3), two additional clusters of residues directly involved in ligand binding have been identified: Cluster 2 (colored in cyan in Figures 1, 2 and 3) that is reasonably conserved and Cluster 3 (colored in orange in Figures 1, 2 and 3) that is quite variable. The contribution of the three clusters to the binding site topology pinpoints to a tripartite architecture (TraR sequence numbering has been taken as reference): i) a conserved core, encompassing residues of Clusters 1 and 2 delimiting the binding site floor and the distal wall (residues 70, 71, 72, 85, 110, 113, 129) shared by QS LuxRs and PAB LuxR solos; ii) a specificity patch, encompassing residues of Clusters 1 and 2 that mainly delimit the binding site roof and the nearby regions of the proximal and distal walls (residues 57, 61, 73, 101, 105) conserved only within the members of the QS LuxRs or the PAB LuxR solos, respectively; iii) a variable part (variability patch), encompassing residues of Cluster 3 delimiting the binding site proximal wall and the nearby regions of the roof and of the floor (residues 49, 53, 58, 62), less conserved even within the members of QS LuxR or PAB LuxR solos respectively.

The detailed molecular cartography of the regulatory domains was extended to the structure-based homology models of the three *Psa* solos, mainly focusing on Clusters 1 and Cluster 2. To this end we have not further discussed Cluster 3 due to its high variability likely to be responsible for the different selectivity towards molecules that belong to the same family of ligands or for the modulation of the degree of “promiscuity” towards members of the same family of compounds. Indeed, the 3D molecular mapping unveils that all of them share the conserved binding site core (marked by ***c*** in Figure 1 and represented in the left column of Figures 2B, 2C and 2D). The prototypes of QS LuxRs (TraR) and of PAB LuxR solos (OryR) are, for comparison, shown in the left column of Figures 2A and 2E respectively.

The identified binding-site molecular determinants of the PsaR1 and PsaR3 regulatory domains suggest a likely shared specificity towards AHL compounds. All the residues defining the specificity patch (marked by ***s*** in Figure 1 and represented in the right column of Figures 2B and 2C for PsaR1 and PsaR3 respectively) indeed differ from those of the PAB LuxR solos subfamily whose prototype OryR is shown in Figure 2E. Interestingly, most of the residues are distinctive of the canonical QS LuxRs whose prototype TraR is shown in Figure 2E. The residues belonging to Cluster 1 and delimiting the roof of the binding site, i.e. PasR1 W69 and PsaR3 W77 (TraR W57) and PsaR1 Y73 and PsaR3 Y81 (TraR Y61) are shared with QS LuxRs, whereas between the two residues, i.e. PsaR3 F119 (TraR F101) and PsaR1 A113, belonging to Cluster 2, only the former is conserved. Moreover, PsaR1 A113 also differs from the highly conserved V116 in PAB LuxR solos.

Similarly, Cluster 2 residues PsaR1 C117 (TraR A105) and PsaR3 R123 are conserved nor in the canonical QS LuxRs nor in the PAB LuxR solos families, the latter being characterized by the occurrence of the highly conserved residue L120. In the distal wall Cluster 2 conserved hydrophobic/aliphatic residues PsaR1 I85 (TraR V73), PsaR3 L93, are substituted by a highly conserved residue Q83 in PAB LuxR solos.

Interestingly, the molecular determinants of the PsaR2 binding site (Figure 2D) closely resemble those of the PAB LuxR solos (Figure 2E), highlighting a different binding specificity, most likely towards plant compounds unrelated to AHLs. Indeed all the residues belonging to the specificity patch are conserved with respect to the PAB LuxR solos subfamily and differ with respect to the canonical QS LuxRs family (Figure 3). The residue PsaR2 and OryR W71, belonging to Cluster 1, among the residues that delimit the roof of the binding site, is highly conserved in all members of the PAB LuxR solos subfamily. The residue TraR Y61 is the corresponding residue that is conversely highly conserved in all members of the QS LuxRs family. Alike, the two PsaR2 and OryR residues V116 and L120 are replaced by the quite conserved TraR F101 and A105 residues respectively. The distal wall residue PsaR2 and OryR Q83, belonging to Cluster 2, is highly conserved in the PAB LuxR solos subfamily, whereas it is substituted by a conserved hydrophobic/aliphatic residue (V/L/M), TraR V73, in QS LuxRs.

### PsaR1, R2 and R3 Solos are Required for *in planta* Survival

**Figure 4:**
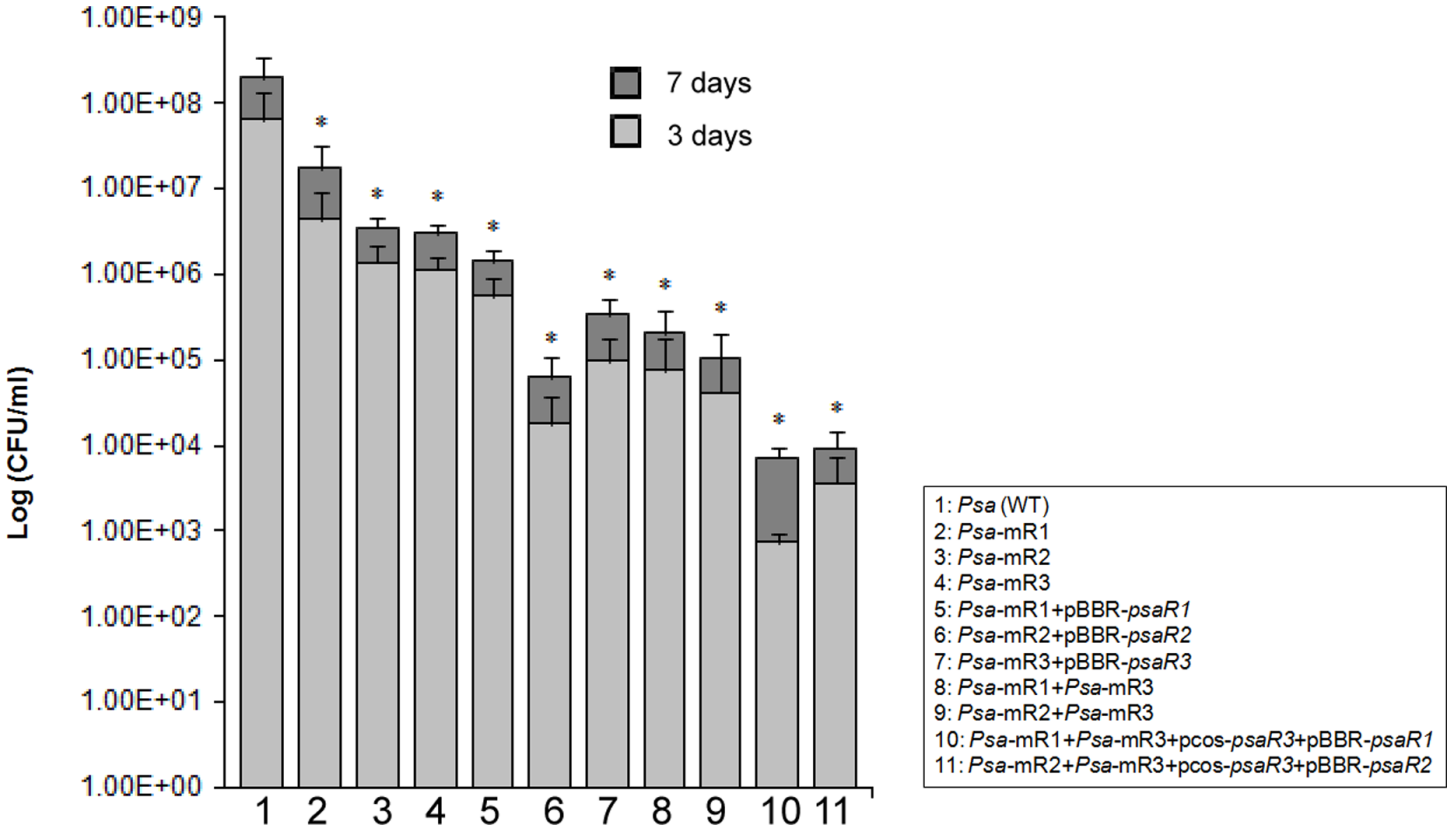
*In planta* survival of *Pseudomonas syringae* pv. *actinidiae* strains in *Actinidia deliciosa* cv Hayward leaf. Histogram reporting *in planta* survival of *Psa* strains: The average bacterial count (log cfu/ml) of three independent experiments is reported with standard deviations for 3^rd^ and 7^th^ day after bacterial inoculation (1–2×10^6^ cfu/ml) in *Actinidia deliciosa* cv. Hayward leaf. Statistical significance with respect to *Psa* wild type is indicated with one asterisk (*P*<0.01).

In order to assess the possible roles of the three *luxR* solos in plant virulence towards kiwifruit, all the three *luxR* solos were mutated creating three independent knock-out mutants. In addition, we have also generated two double mutants having two of the solos inactivated; namely the *psa-*mR1*+psa-*mR3 and *psa-*mR2*+psa-*mR3 double mutants. All these mutants were inoculated on *A. deliciosa* and *A. chinosa* kiwifruit leaves and bacterial multiplication and survival was determined after the 3^rd^ and 7^th^ day after inoculation by bacterial count (cfu/ml). All the three *luxR* solos mutants were found significantly impaired in *in planta* survival and multiplication compared to wild type *Psa* (Figure 4). Cfu/ml for *psaR1* mutant was approximately 10 fold less compared to wild type level on the 3^rd^ and 7^th^ day after infection. The cfu/ml count for *psaR2* and *psaR3* mutants were found at least 100 fold less than the wild type level. The solo double mutants showed a further reduction compared to respective *luxR* solo single mutants. Compared to wild type the double mutants showed at least 1000 fold less cfu/ml count in the kiwifruit leaf after 3^rd^ and 7^th^ day of observation. These results implicate the three solos as being important for *in planta* growth and multiplication. We did not observe the recovery of these *in planta* survival phenotype upon complementation by providing the wild-type gene *in trans* in a plasmid on the single and double mutated *luxR* solos (Figure 4). We do not know the reason for this, however it has been previously observed that over-expression of this sub-family of *luxR* solos can have unexpected phenotypes and do not result in recovery of phenotypes [37], [43].

### *psaR1, psaR2* and *psaR3* are not Auto-regulated and *psaR2* does not Regulate *pip in vitro*

In order to study the expression and possible auto-regulation of the three *luxR* solos, we cloned the promoter of *psaR1*, *psaR2* and *psaR3* genes in a promoter probe vector harboring a promoterless *lacZ* gene. Gene promoter studies revealed that *psaR2* was highly expressed compared to the other solos and that the three genes are not autoregulated under the conditions we tested (Table 3). As mentioned above, PsaR2 belongs to the sub-family of solos found in many plant associated bacteria (PAB) and which respond to yet unknown plant signal molecules [35]. All members of this LuxR solo sub-family contain an adjacent proline iminopeptidase (*pip*) gene which is regulated by the solo. We cloned the *pip* promoter in promoter probe vector and introduced it into the wild type and *psaR2* mutant. We performed β-galactosidase assay in the presence and absence of plant kiwifruit leaf macerate extract; no significant increase in *pip* gene expression was observed in the presence of the plant extract (Table 4). Induction of *pip* gene expression is not always possible via the solos using plant extracts as the signal molecule might not be present in large amounts in the particular tissue and/or growth stage of the plant.

**Table 3:** Expression and auto-regulation of *psaR1, psaR2 and psaR3.*

Strains	Average Miller unit	Standard deviation
*Psa* (WT)+pMP-*psaR1*	54.99^a^	1.42
*Psa*-mR1*+*pMP-*psaR1*	47.15^a^	2.29
*Psa* (WT)+pMP-*psaR2*	759.06^b^	13.23
*Psa*-mR2*+*pMP-*psaR2*	769.00^b^	19.38
*Psa* (WT)+pMP-*psaR3*	60.85^a^	4.41
*Psa*-mR3*+*pMP-*psaR3*	64.44^a^	0.29

Statistical analyses (Student’s *t* test) were performed to compare the significance difference in promoter activity between wild type *Psa* strain and respective mutants. a, Not significant difference to a at *P*<0.05; b, significant difference to a at *P*<0.001 but not significant difference to b at *P*<0.05.

**Table 4:** Expression and regulation of *psa-pip.*

Media	Strains	Average Miller unit	Standard deviation
KB	*Psa* (WT)+pMP-*psa-pip*	91.75^a^	2.21
KB+Kiwi	*Psa* (WT)+pMP-*psa-pip*	126.82^c^	7.68
KB	*Psa*-mR2+pMP*-psa-pip*	103.60^b^	3.47
KB+Kiwi	*Psa*-mR2*+*pMP-*psa-pip*	122.62^c^	7.56

Expression of *psa-pip* was assessed in presence and absence of kiwi leaf extract for wild type (WT) and *psaR2* mutant (*Psa-*mR2). Statistical analyses (Student’s *t* test) were performed to compare the significant difference in promoter analysis between wild type *Psa* strain and *Psa*-mR2 mutant in the presence and absence of kiwi extract. a, significant difference to b at *P*<0.01 and significant difference to c at *P*<0.05. c, not significant difference to c at *P*<0.05 but significant difference to a and b at *P*<0.05.

### Response of PsaR1 and PsaR3 to AHLs

**Figure 5:**
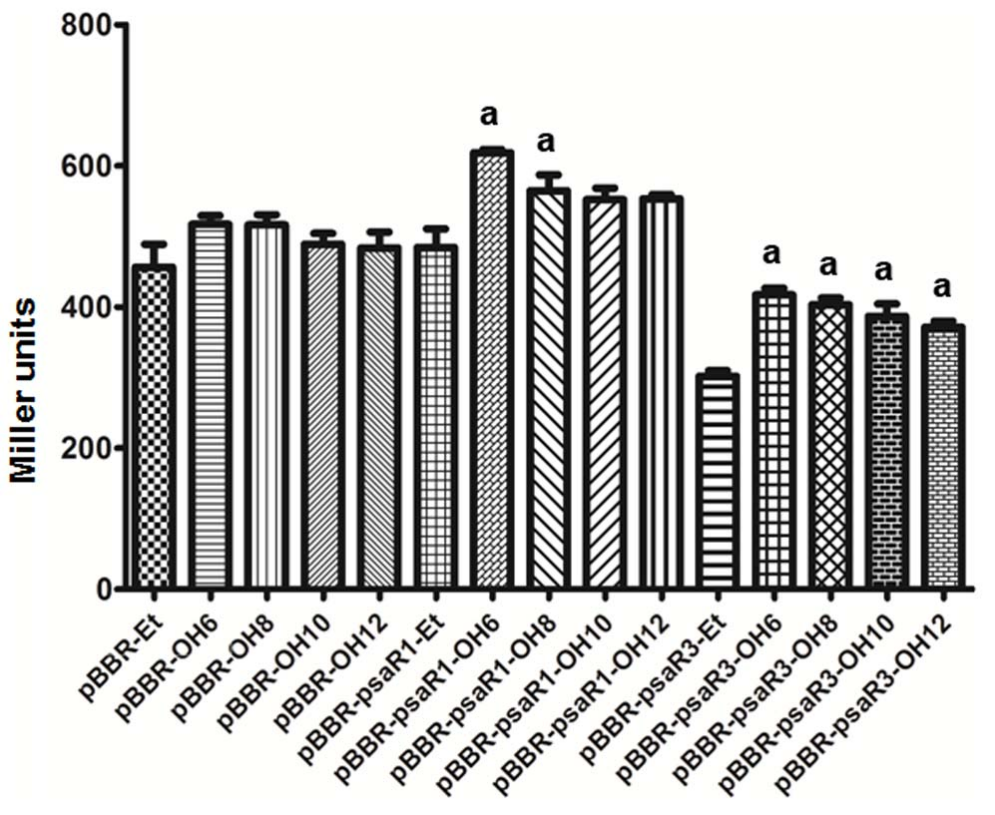
Response of PsaR1 and PsaR3 to AHLs. Histogram reporting gene promoter activity of *E. coli* harboring pMULTIAHLPROM in the presence of either pBBR, pBBR-*psaR1* and pBBR-*psaR3* plasmids and different hydroxy AHLs (OH-C6-AHL, OH-C8-AHL, OH-C10-AHL and OH-C12-AHL). All measures were performed in biological triplicates, and the mean Miller units with standard deviations are shown. Promoter activity was statistically significantly (*P*<0.05) increased (a) in the presence of OH-C6-AHL and OH-C8-AHL for PsaR1 compared to respective pBBR empty vector control. Similarly, for PsaR3 statistically significantly (*P*<0.05) increase (a) in *lacZ* expression was observed in the presence of OH-C6-AHL, OH-C8-AHL, OH-C10-AHL and OH-C12-AHL compared to respective pBBR empty vector controls.

In order to test if PsaR1 and PsaR3 respond to any AHL signals, we performed an assay in *E. coli* harboring the pMULTIAHLPROM plasmid carrying a synthetic tandem promoter of seven different *luxI* gene promoters transcriptionally fused to a promoterless *lacZ* which respond to several different LuxR family proteins [70]. We introduced the pBBR empty vector as well as pBBR constructs containing either *psaR1* or *psaR3* in *E. coli* (pMULTIAHLPROM) and determined *lacZ* activities providing many structurally different exogenous AHLs. If PsaR1 and PsaR3 bind an AHL and recognize at least one of the promoters in pMULTIAHLPROM, this will result in an increase in *lacZ* activity. We analyzed all the structurally different AHLs having C4-12 acyl chains, unsubstituted at position C3 or having a ketone or a hydroxy. Results show that promoter activity was statistically significantly (*P*<0.05) increased only in the presence of OH-C6-AHL (28% increase) and OH-C8-AHL (16% increase) for PsaR1 and in the presence of OH-C6-AHL (38% increase), OH-C8-AHL (34% increase) OH-C10-AHL (28% increase) and OH-C12-AHL (23% increase) for PsaR3 compared to the same growth conditions in the absence of added exogenous AHLs and to the empty vector control (Figure 5). The background promoter activity that we observed could be to the SdiA solo present in *E. coli* which is known to respond to several AHLs [31]. In summary, we have detected a response if PsaR1 and PsaR3 respond to AHLs, future studies needs to involve biochemical analysis and identification of potential target gene(s).

### Phenotypic Studies on the Three LuxR Solos

It was of interest to determine if any of the three LuxR solos were involved in regulating phenotypes which are known to be relevant to bacterial communities and potentially important for virulence. Bacterial movement via swarming and swimming was tested as described in the Materials and Methods section. Mutants of *psaR1* and *psaR2* swarmed just like the wild type whereas the *psaR3* swarmed less (Table 5). The *psaR1-psaR3* and *psaR2-psaR3* double mutants also displayed a reduced swarming phenotype (Table 5). These results indicate that *psaR3* is involved in regulating swarming in *Psa*. Complementation experiments of *psaR3* and the two double mutants using the wild type *psaR3* gene harboured in a plasmid resulted in a more pronounced defect in swarming. The reason for this is not known, it is possible that extra copies of the regulator might result in causing this defect in swarming. A very similar trend of the role of *psaR3* was observed when testing its role in swimming (Table 5). In order to test resistance to oxidative stress we tested all *Psa luxR* solo mutants for H_2_O_2_ sensitivity on a plate assay as described in the Materials and Methods section. The three single solo mutants displayed similar resistance to H_2_O_2_ as the wild type whereas the two double mutants were found to be more sensitive to H_2_O_2_ as they both formed a visually larger clearing zone compared to wild type. Complemented strains were also found more sensitive to H_2_O_2,_ particularly the over expression of *psaR1* in double mutant background was found more drastically affected for H_2_O_2_ sensitivity (data not shown). We also tested *Psa* for several secreted enzyme activities and have detected lipase activity; results from plate assay indicated that *psaR1* and *psaR2* do not affect lipase activity whereas the *psaR3* mutant resulted in a decrease of lipase secretion compared to wild type *Psa*. The double mutant in *psaR1* or *psaR2* in the *psaR3* mutant background were also decreased for the lipase activity. Complementation of *psaR3* did not restore the phenotype whereas *psaR1* and *psaR2* expression *in trans* increased the lipase secretion in plate assay (Table 6, Figure S1). The AHL independent role of these solos in controlling these phenotypes can be due to background activity or possible a negative effect of AHLs. In the future performing the same experiments in the presence of AHLs will help to determine role of AHLs.

**Table 5:** Swarming and swimming movement score of *Pseudomonas syringae* pv. *actinidiae* strains.

Psa Strains	Swarming (0.8% KBA)Average ± S.D. in mm	Swarming (0.6% KBA)Average ± S.D. in mm	Swimming 0.3% KBA)Average ± S.D. in mm
*Psa* (WT)	65.33±1.5	80.00±0.5	66.50+/−5.8
*Psa*-mR1	64.00±1.48	75.00±3.13	65.00±5.22
*Psa*-mR2	63.00±1.04^a^	77.50±2.71	65.50±3.73
*Psa*-mR3	55.00±1.04^a^	72.00±2.09^a^	37.50±7.83^a^
*Psa*-mR1+pBBR-*psaR1*	54.33±0.49^a^	79.00±1.04	62.00±2.95
*Psa*-mR2+pBBR-*psaR2*	50.00±1.20^a^	77.00±2.33	59.00±1.04
*Psa*-mR3+pBBR-*psaR3*	37.00±0.60^a^	72.50±2.71^a^	37.50±7.96^a^
*Psa*-mR3+*Psa*-mR1	30.25±0.62^a^	69.50±3.92^a^	33.00±3.13^a^
*Psa*-mR3+*Psa*-mR2	30.58±0.80^a^	65.50±1.50^a^	27.00±3.14^a^
*Psa*-mR3+*Psa*-mR1+ pcos-*psaR3*+ pBBR-*psaR1*	7.50±0.522^a^	10.50±0.90^a^	29.00±1.04^a^
*Psa*-mR3+*Psa*-mR2+ pcos-*psaR3*+ pBBR-*psaR2*	21.00±1.04^a^	57.50±2.61^a^	40.00±4.35^a^

Mean values and standard deviations were calculated for swarming and swimming bacterial movement obtained from three replications each on 0.8%, 0.6% and 0.3% KBA. Statistical analyses (Student’s *t* test) were performed to compare the significant difference in bacterial movement between wild type *Psa* strain and mutated and complemented strains. a, significant difference to WT at *P*<0.0001.

**Table 6:** Lipase secretion score of *Pseudomonas syringae* pv. *actinidiae* strains.

Psa Strains	Lipase secretion score in LB Agar-tributyrinplate Average ± S.D. in mm
*Psa* (WT)	4.00±0.00
*Psa*-mR1	3.83±0.29
*Psa*-mR2	3.83±0.29
*Psa*-mR3	2.83±0.29^a^
*Psa*-mR1+pBBR-*psaR1*	4.25±0.25
*Psa*-mR2+pBBR-*psaR2*	4.17±0.29
*Psa*-mR3+pBBR-*psaR3*	2.00±0.00^a^
*Psa*-mR3+*Psa*-mR1	2.50±0.00^a^
*Psa*-mR3+*Psa*-mR2	2.50±0.00^a^
*Psa*-mR3+*Psa*-mR1+ pcos-*psaR3*+ pBBR-*psaR1*	4.83±0.29^ab^
*Psa*-mR3+*Psa*-mR2+ pcos-*psaR3*+ pBBR-*psaR2*	3.17±0.29^a^

Mean values and standard deviations were calculated for halo obtained from three replications of lipase secretion in LB Agar-tributyrin plates. Statistical analyses (Student’s *t* test) were performed to compare the significant difference in lipase secretion between wild type *Psa* strain and mutated and complemented strains. a, significant difference to WT at *P*<0.05. b, significant difference to ‘a’ at *P*<0.01.

## Concluding Remarks

The emerging pathogen *Pseudomonas syringae* pv. *actinidiae* (*Psa*) causing bacterial canker of kiwifruit crops has been isolated in several countries and genome based comparison studies of many isolates had been suggested the presence of variations among different strains [49], [59]–[61]. Analysis of the genome and experiments presented here lead to the conclusion that a canonical AHL LuxI/R QS system is absent in *Psa*. We found, however, that *Psa* possesses three LuxR solos; two of these could possibly be binding to AHLs, whereas one was found to belong to a sub-family of plant associated bacteria (PAB) solos which responds to yet unidentified plant signals [35], [41]. The genetic inactivation of these three putative *luxR* solos (*psaR1*, *psaR2 psaR3*) either alone or in combination of double mutation affected *in planta* survival implicating them in *in planta* fitness. The PsaR3 solo was found to be involved in motility and lipase production.

The PAB PsaR2 solo is most likely involved in responding to a plant signal and ortholog proteins in *Xanthomonas, Pseudomonas* and *Sinorhizobium* have been shown to be regulating plant-associated traits. It is therefore likely that this interkingdom system is also involved in regulating genes in *Psa* implicated in virulence, growth or persistence in the kiwifruit plant.

In summary, this study shows that *Psa* possesses three LuxR solos which is rather unusual as most commonly proteobacteria possess only one. The PsaR2 solo is most likely involved in interkingdom signaling whereas PsaR1 and PsaR3 could be responding to exogenous AHLs produced by neighboring bacteria. It must be kept in mind however that very few LuxR solos have been studied and they could be involved in responding to other types of signals. For example a recent study has reported that a LuxR solo from *Photorhabdus luminescens* responds to an endogenous signal which is not an AHL [71] thus it cannot be excluded that PsaR1 and PsaR3 be part of a novel QS system involving new types of signals.

## Materials and Methods

### Bacterial Strains, Media and Culture Conditions

The bacterial strains used in this study are listed in Table 1. *Escherichia coli* DH5α [72] was grown in LB medium at 37°C. *Agrobacterium tumefaciens, C. violaceum* CV026 and *E. coli* (pSB401) biosensors [70], [73] were grown as recommended. *Pseudomonas syringae* pv. *actinidiae* (*Psa*) was grown either in LB medium, KB medium or NSA medium at 25°C (room temperature). The following antibiotic concentrations were used: Nitrofurantoin (Nf) 150 µg/ml; ampicillin (Amp) 100 µg/ml; kanamycin (Km) 100 µg/ml; tetracycline (Tc) 10 µg/ml (*E. coli*), 40 µg/ml (*Psa*); gentamycin (Gm) 10 µg/ml (*E. coli*), 40 µg/ml (*Psa and A. tumefaciens*).

### Recombinant DNA Techniques

Plasmids used or generated in this study and details on their construction are listed in Table S1. Routine DNA manipulation steps such as digestion with restriction enzymes, agarose gel electrophoresis, purification of DNA fragments, ligations with T4 ligase, radioactive labeling by random priming and transformation of *E. coli* etc. standard procedures were performed as described previously [74]. Colony hybridizations were performed using N+Hybond membrane (Amersham Biosciences); plasmids were purified using the EuroGold plasmid columns (Euro Clone) or with the alkaline lysis method [75]; total DNA from *Pseudomonas* strains were isolated by Sarkosyl/Pronase lysis as described previously [76]. PCR amplifications were performed using Go-Taq DNA polymerase or pfu DNA polymerase (Promega). The oligonucleotide primers used in this study are listed in Table S2. Automated sequencing was performed by Macrogen sequence service (Europe). Triparental matings between *E. coli* and *Psa* were carried out with the helper strain *E. coli* DH5α (pRK2013) [77].

### AHLs Extraction and Detection

Culture supernatant extracts of *Psa* strains were analyzed on C_18_ reverse-phase TLC plates as described previously [78]. In brief, different isolates of *Psa* strains were grown in KB medium at room temperature. 30 hrs grown 50 ml cultures were pelleted down and supernatants were further extracted with similar volume of ethyl acetate containing 0.1% acetic acid by vortexing. The lower organic phase was discarded and upper watery phase was transferred to a glass beaker and dried over-night in a laminar hood. Dried AHLs in glass beaker were then dissolved in the 10 ml of same extraction solvent using magnetic stirrer. The dissolved AHLs were further concentrated to a final volume of 100–200 µl using vacuum dryer. Extraction debris were removed by pelleting at 13000 rpm for two minutes and clear extracts were loaded onto a pre warmed TLC sheet and run by 70% methanol. After completion of the TLC run, it was dried in laminar hood and was then overlaid with a thin layer of AB top agar seeded either with *A. tumefaciens* NTL4/pZLR4 in the presence of X-Gal (100 µg/ml), as described previously [78], or Luria-Bertani top agar seeded with *C. violaceum* CVO26 [79] or *E. coli* pSB401 [80].

### Bioinformatic Search for *luxR* Solos and their Analysis

We looked for the genes annotated as LuxR in the draft genome of *Psa*. All the LuxR sequences obtained in genome search were further analysed for autoinducer binding domain at conserved domain database (http://www.ncbi.nlm.nih.gov/Structure/cdd/wrpsb.cgi). Three LuxR sequences with autoinducer binding domain that were selected and used in this study have been designated as PsaR1, PsaR2 and PsaR3. We retrieved the protein sequences of known LuxR solos from several plant associated bacteria using PUBMED and aligned them using Clustal Omega service available at http://www.ebi.ac.uk/Tools/msa/clustalo/. Multiple aligned LuxR solos were further exported as tiff file and edited for domains and highlighting the key amino acid residues. A phylogeny was also generated for these aligned sequences at Clustal Omega service.

### Homology Modeling and Structural Alignments

Three dimensional structure-based homology models were built using I-TASSER [65]. The top-scored models (with C-scores of 0.60, of 0.78 and −0.45 respectively) were based on TraR from *Sinorhizobium fredii* [PDB_ID 2Q0O] [66] for PsaR1 and on QscR from *Pseudomonas aeruginosa* [PDB_ID 3SZT] [67] for both PsaR2 and PsaR3 and were validated by two complementary protein model quality predictors. The correctness of the selected models was assessed by ProQ [81] and exploiting PSIPRED [82] for secondary structure prediction, resulting in the predicted LGscores and MaxSub values of 3.542, 3.897 and 3.339, and 0.451, 0.362 and 0.368, respectively. The overall quality of the models obtained was validated by a neural network approach using AIDE [83], the statistical indicators TM-score and RMSD being 0.63, 0.67 and 0.72, and 6.63 Å, 5.36 Å and 4.88 Å, respectively.

Sequence alignment was performed by Expresso [84] that exploits structural aligners algorithms like SAP [85] or TMalign [86] to generate structure-based alignments of the templates, used to obtain the structure-based homology models, and TraR from *Agrobacterium tumefaciens* (PDB_ID 1H0M [68], the prototype of canonical QS LuxR family. The achieved score (the total consistency value) of 97 is highly reliable, being 100 the full agreement between the considered alignment and its associated primary library that has been computed as a first step of the consistency-based protocol exploited by Expresso. Then, the structure-based homology model of OryR from *Xanthomonas oryzae* [69], a prototype of PAB LuxR solos, and the three structure-based homology models of LuxR solos from *Psa* were structurally aligned based on the secondary structure prediction according to I-TASSER [65].

### Construction of *Psa luxR* Solos Mutants

The psaR3 in frame deletion mutant was generated using the pEX19Gm plasmid as described previously [87]. Briefly, deleting the internal region (249 bp) of *psaR3* gene, two external fragments; Frag1 (527 bp) and Frag2 (539 bp) were PCR amplified using primers listed in Table S2 and sequentially cloned in pEX19Gm as mentioned in Table S1. The resulting pEX19Gm-derivative plasmid, listed in Table S1, was introduced in Psa *10,22* by conjugation. Clones with a chromosomal insertion of the pEX19Gm plasmids were selected on LB agar plates supplemented with 50 µg/ml Gm and 150 µg/ml Nf. Plasmid excision from the chromosome was subsequently selected on LB agar plates supplemented with 10% (w/v) sucrose. The *psaR1* and *psaR2* mutants were generated using plasmid integration by pKNOCK-Km suicide delivery system. Briefly, an internal fragment of *psaR1* (372 bp) and *psaR2* (390 bp) were PCR amplified by using primers listed in Table S2 and sequentially cloned in pKNOCK-Km yielding pKNOCK-*psaR1* and pKNOCK-*psaR2* as mentioned in Table S1. pKNOCK-*psaR1* and pKNOCK-*psaR2* plasmids were further used as a suicide delivery system and *psaR1* and *psaR2* mutants were created as previously described [88]. *Psa* mutant strains were verified by PCR analysis and sequencing.

### Complementation of *Psa luxR* Solo Mutants

We PCR amplified the full length *psaR1*, *psaR2* and *psaR3* genes using primers listed in Table S2 and cloned in the pBBR-Gm vector [89] as mentioned in Table S1. pBBR plasmids containing full length *luxR* solo genes, pBBR-*psaR1*, pBBR-*psaR2* and pBBR-*psaR3* (Table S1), were introduced in mutants *Psa*-mR1, *Psa*-mR2 and *Psa*-mR3 respectively by conjugation. Positive clones were selected on LBA plates supplemented with 50 µg/ml Gm, 50 µg/ml Km and 150 µg/ml Nf.

In order to complement the double mutants, a cosmid library was constructed of *Psa* 10,22 strain by using the cosmid pLAFR3 [90] as vector. Insert DNA was prepared by partial EcoRI digestion of the genomic DNA and then ligated into the corresponding site in pLAFR3. The ligated DNA was then packaged into λ phage heads using Gigapack III Gold packaging extract (Stratagene) and the phage particles were transduced to *E. coli* HB101 as recommended by the supplier. In order to identify the cosmid containing the *luxR* genes, the cosmid library was screened using full length *psaR3* gene as a radiolabelled probe in colony hybridization. We obtained a cosmid clone containing *psaR3* (pcos-*psaR3*) and was harbored together with a pBBR clone containing one of the other *luxR* solos (i.e. pBBR-*psaR1* and pBBR-*psaR2*; Table S1). In this way *Psa*-mR3+*Psa*-mR1 and *Psa*-mR3+*Psa*-mR2 double mutants were complemented.

### β-galactosidase Activity, Lipase, Motility and H_2_O_2_ Sensitivity Assay

The *psaR1*, *psaR2*, *psaR3* and *pip* gene promoter regions were PCR amplified using primers listed in Table S2 and cloned into promoter probe vector pMP220 which harbours a promoterless *lacZ* gene as described in Table S1. pMP-*psaR1*, pMP-*psaR2* and pMP-*psaR3* were then introduced independently into the WT and derivative *Psa*-mR1, *Psa*-mR2 and *Psa*-mR3 mutants by conjugation. pMP-*pip* was introduced only in the WT and in the *Psa*-mR2 mutant. β-galactosidase assays were performed as previously described [91]. Average Miller unit values and standard deviations were calculated from three independent experiments.

Lipase secretion phenotype for *Psa* strains were performed as mentioned previously with some modifications [92]. Briefly, for plate assays 1 ml of tributyrin solution was added to a 10 ml of LB broth and sonicated using a sonicator (four pulse of 60–80 Hrtz) until the solution to become homogenous white. This homogenous tributyrin mix was added to pre-warmed 400 ml of LB Agar media, mixed well and poured onto petriplates. All the *Psa* strains grown for 36 hrs were harvested washed with LB media and adjusted to OD 1.0 at OD_600_. 1 µl of equalized *Psa* strains were spotted onto dried LB Agar-tributyrin plates and incubated at room temperature for further periodical observation. Plates were scanned at 7^th^ day and lipase halo data were scored. Mean values and standard deviations were calculated and statistical analysis were performed for three replicates.

For bacterial motility assays, all the *Psa* strains were grown in KB broth for 24 hrs at room temperature and adjusted to OD = 1.0. 2 µl of adjusted cultures were spotted onto 0.5 mm filter disc placed in the centre of 0.6% and 0.8% KB agar plates for swarming motility. Similarly for swimming motility 2 µl of adjusted cultures were spotted directly in the centre of 0.3% KB agar plates. The plates were incubated at room temperature and the diameter of swarming and swimming were measured in three dimensions after 24 hrs and 48 hrs and the mean values were calculated. All the experiments were performed in triplicate and the mean values and standard deviations are presented.

In order to measure the H_2_O_2_ sensitivity, *Psa* strains grown in KB broth at room temperature were adjusted to OD = 1.0. 100 µl of adjusted bacterial culture were added to 25 ml of pre warmed 0.6% KBA, mixed well and poured on petri plates. Four microliter of 33.3% H_2_O_2_ was pipetted onto 3 MM Whatman paper disks (0.5 cm diameter) and these disks were placed in the centre and on top of the bacterial plates and incubated at room temperature overnight. The zone of bacterial inhibition, in mm, was taken as a measure of H_2_O_2_ sensitivity. Plates were scanned and zone of inhibition was measured in three dimensions and the mean values, standard deviations and statistics were calculated from three independent replications.

### AHL Response to QS LuxR Solos

In order to assess if QS LuxR solos PsaR1 and PsaR3 respond to AHLs, we performed promoter activity of *E. coli* harboring pMULTIAHLPROM [70] in the presence of either PsaR1 or PsaR3 and different AHLs. Briefly, pBBR, pBBR-*psaR1* and pBBR-*psaR3* plasmids (Table S1) were introduced into *E. coli* (pMULTIAHLPROM). β-galactosidase activity was performed for these strains in the presence of different AHL molecules and ethyl acetate as a baseline control. Average Miller unit values and standard deviations were calculated from three independent experiments.

### *In planta* Survival Assay

*Psa in planta* survival assay was performed as described previously [49]. For the survival assay, one-year-old, potted plants of *A. deliciosa* cv. Hayward were used. The plants were maintained in a climatic room and watered regularly. For inoculation, *Psa* strains were grown for 48 hrs on NSA medium supplemeted with antibiotics, at 23–25°C. Bacterial culture were pelleted down washed with sterile saline (0,85% NaCl in distilled water) and adjusted to 1–2×10^6^ cfu/ml in sterile saline. Leaf areas of approximately 1 cm in diameter were inoculated using a needleless sterile syringe with the bacterial suspension. For each strain, 10 leaves were inoculated in four sites and control plants were treated in the similar manner using sterile saline. In order to determine *in planta* bacterial growth, leaf disks of about 0.5 cm of diameter were sampled from inoculation site at 3rd and 7th days post inoculation, ground in 1 ml of sterile saline, and serial ten-fold dilutions were plated onto NSA supplemented with antibiotics. Colonies were counted two days after incubation at 23–25°C. Cfu/ml determined for each strain were plotted as log values on excel graph. Confirmation of colony identity was achieved by following well established procedures [48], [49], [93].

## Supporting Information

Figure S1 Lipase secretion in LB Agar-tributyrin plate assay. Figure showing lipase secretion phenotype on LB Agar-tributyrin plate for *Psa* strains. (1) Wild type, (2) *psa*-mR1, (3) *psa*-mR2, (4) *psa*-mR3, (5) *psa*-mR1+pBBR-*psaR1*, (6) *psa*-mR2+pBBR-*psaR2*, (7) *psa*-mR3+pBBR-*psaR3*, (8) *psa*-mR1+*psa*-mR3, (9) *psa*-mR2+*psa*-mR3, (10) *psa*-mR1+*psa*-mR3+pcos-*psaR3*+pBBR-*psaR1*, (11) *psa*-mR2+*psa*-mR3+pcos-*psaR3*+pBBR-*psaR2.*
(TIF)

Table S1 Plasmids used in this study.
(DOC)

Table S2 List of oligonucleotide primers used in this study.
(DOC)

Table S3 Flanking genes to *psaR1*, *psaR2* and *psaR3* in *Pseudomonas syringae* pv. *Actinidiae*.
(DOC)
