# Supplementary figures and images for: The Kiwifruit Emerging Pathogen Pseudomonas syringae pv. actinidiae Does Not Produce AHLs but Possesses Three LuxR Solos
Source: PLoS One. 2014 Jan 31;9(1):e87862. doi: 10.1371/journal.pone.0087862 (PMC3909224; doi:10.1371/journal.pone.0087862)

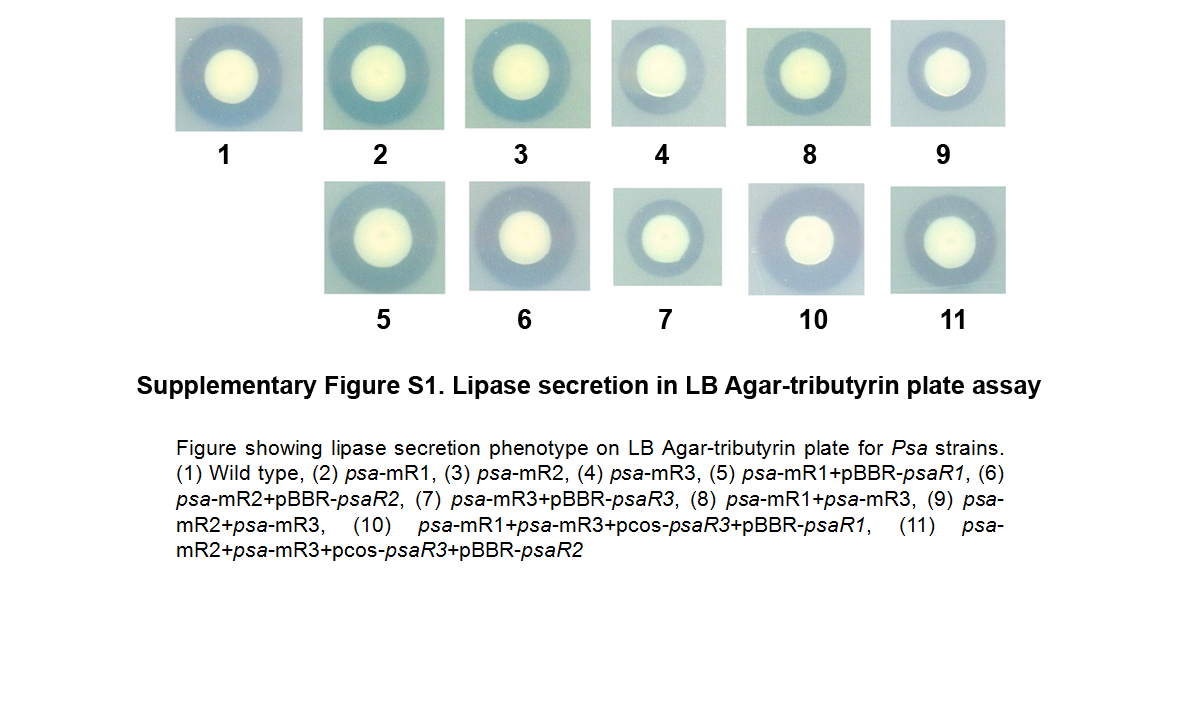

Supplement: Figure S1 — Lipase secretion in LB Agar-tributyrin plate assay. Figure showing lipase secretion phenotype on LB Agar-tributyrin plate for Psa strains. (1) Wild type, (2) psa-mR1, (3) psa-mR2, (4) psa-mR3, (5) psa-mR1+pBBR-psaR1, (6) psa-mR2+pBBR-psaR2, (7) psa-mR3+pBBR-psaR3, (8) psa-mR1+psa-mR3, (9) psa-mR2+psa-mR3, (10) psa-mR1+psa-mR3+pcos-psaR3+pBBR-psaR1, (11) psa-mR2+psa-mR3+pcos-psaR3+pBBR-psaR2. (TIF) [file pone.0087862.s001.tif]
