# Supplementary material for: The Kiwifruit Emerging Pathogen Pseudomonas syringae pv. actinidiae Does Not Produce AHLs but Possesses Three LuxR Solos
Source: PLoS One. 2014 Jan 31;9(1):e87862. doi: 10.1371/journal.pone.0087862 (PMC3909224; doi:10.1371/journal.pone.0087862)
Supplement: Table S1 — Plasmids used in this study. (DOC) [file pone.0087862.s002.doc]

**Table S1.** Plasmids used in this study

| **Plasmids** | Relevant characteristics and plasmid construction | **Reference/ Source** |
| --- | --- | --- |
| pGEM-T Easy | Cloning vector; Amp^r^ | Promega |
| pMP220 | Promoter probe vector, IncP; Tet^r^ |  |
| pKNOCK-Km | Conjugative suicide vector; Km^r^ |  |
| pBBR1MCS-5 | Broad-host-range vector; Gm^r^ |  |
| pBluscript KS | Cloning vector; Amp^r^ | Stratagene |
| pLAFR3 | Broad-host-range cloning vector, IncP1; Tet^r^ |  |
| pRK2013 | Tra^+^ Mob^+^ ColE1 replicon; Km^r^ |  |
| pEX19Gm | *oriT*^+^*sacB*^+^, gene replacement vector with MCS from pUC18; Gm^r^ |  |
| pMULTIAHLPROM | Tet^r^ |  |
| pGEM-*psaR1*int | An internal fragment of *psaR1* (372 bp) was PCR amplified by using luxR1-Pkn-F and luxR1-Pkn-R primers (Table S2) and cloned into pGEM | This study |
| pGEM-*psaR2*int | An internal fragment of *psaR2* (390 bp) was PCR amplified by using luxR2-Pkn-F and luxR2-Pkn-R primers (Table S2) and cloned into pGEM | This study |
| pGEM-*psaR3*-Fr1 | Deleting the internal region (249 bp) of *psaR3*gene, an upstream fragment; Frag1 (527 bp) was PCR amplified using Pr EXR3 act Kpn/Pr EXR3 act Hn1 primers (Table S2) and cloned into pGEM | This study |
| pGEM-*psaR3*-Fr2 | Deleting the internal region (249 bp) of *psaR3*gene, a downstream fragment; Frag2 (539 bp) was PCR amplified using Pr EXR3 act Hn2/Pr EXR3 actXba primers and cloned into pGEM | This study |
| pGEM-*psaR1*-Fl | Full length *psaR1* gene including some upstream and downstream region were PCR amplified using pBR R1 act Kpn+pBR R1 Xba primers (Table S2) and cloned into pGEM | This study |
| pGEM-*psaR2*-Fl | Full length *psaR2*gene including some upstream and downstream region were PCR amplified using pBR R2 act Kpn+pBR R2 Xba primers (Table S2) and cloned into pGEM | This study |
| pGEM-*psaR3*-Fl | Full length *psaR3*gene including some upstream and downstream region were PCR amplified using pBR R3 act Kpn+pBR R3 Xba primers (Table S2) and cloned into pGEM | This study |
| pGEM-*psaR1*-Pr | Promoter region of *psaR1*was PCR amplified using pr-R1-Bam and pr-R1-Xho primers (Table S2) and cloned into pGEM | This study |
| pGEM-*psaR2*-Pr | Promoter region of *psaR2*was PCR amplified using pr-R2-Bam and pr-R2-Xho primers (Table S2) and cloned into pGEM | This study |
| pGEM-*psaR3*-Pr | Promoter region of *psaR3*was PCR amplified using pr-R3-Bam and pr-R3-Xho primers (Table S2) and cloned into pGEM | This study |
| pGEM-*psa*-*pip*-Pr | Promoter region of *psa*-*pip* gene was PCR amplified using pr-pip-Bam and pr-pip-Xho primers (Table S2) and cloned into pGEM | This study |
| pKNOCK-*psaR1* | An EcoR1 digested fragment from pGEM-*psaR1*int was cloned into EcoR1digested pKNOCK-Km | This study |
| pKNOCK-*psaR2* | An EcoR1 digested fragment from pGEM-*psaR2*int was cloned into EcoR1digested pKNOCK-Km | This study |
| pBluescript-*psaR3*-Fr1 | KpnI and HindIII digested fragment from pGEM-*psaR3*-Fr1 was cloned into KpnI and HindIII digested pBluescript | This study |
| pBluescript-*psaR3*-Fr1+2 | HindIII and XbaI digested fragment from pGEM-*psaR3*-Fr2 was cloned into HindIII and XbaI digested pBluescript-*psaR3*-Fr1 | This study |
| pEX-*psaR3*-Fr1+2 | KpnI and XbaI digested fragment from pBluescript-*psaR3*-Fr1+2 was cloned into KpnI and XbaI digested pEX19Gm | This study |
| pcos-*psaR3* | Cosmid clone containing full length *psaR3* gene | This study |
| pBBR-*psaR1* | KpnI and XbaI digested fragment from pGEM-*psaR1*-Fl was cloned into KpnI and XbaI digested pBBR-Gm^r^ plasmid | This study |
| pBBR-*psaR2* | KpnI and XbaI digested fragment from pGEM-*psaR2*-Fl was cloned into KpnI and XbaI digested pBBR-Gm^r^ plasmid | This study |
| pBBR-*psaR3* | KpnI and XbaI digested fragment from pGEM-*psaR3*-Fl was cloned into KpnI and XbaI digested pBBR-Gm^r^ plasmid | This study |
| pMP-*psaR1* | BamHI and XhoI digested fragment from pGEM-*psaR1*-Pr  was cloned into BamHI and XhoI digested pMP220 plasmid | This study |
| pMP-*psaR2* | BamHI and XhoI digested fragment from pGEM-*psaR2*-Pr  was cloned into BamHI and XhoI digested pMP220 plasmid | This study |
| pMP-*psaR3* | BamHI and XhoI digested fragment from pGEM-*psaR3*-Pr  was cloned into BamHI and XhoI digested pMP220 plasmid | This study |
| pMP-*psa*-*pip* | BamHI and XhoI digested fragment from pGEM-*psa*-*pip*-Pr  was cloned into BamHI and XhoI digested pMP220 plasmid | This study |
