# Supplementary material for: The Kiwifruit Emerging Pathogen Pseudomonas syringae pv. actinidiae Does Not Produce AHLs but Possesses Three LuxR Solos
Source: PLoS One. 2014 Jan 31;9(1):e87862. doi: 10.1371/journal.pone.0087862 (PMC3909224; doi:10.1371/journal.pone.0087862)
Supplement: Table S2 — List of oligonucleotide primers used in this study. (DOC) [file pone.0087862.s003.doc]

**Table S2. List of oligonucleotide primers used in this study**

| **Primer Name** | **Sequence (5’ to 3’)** | **Reference/ Source** |
| --- | --- | --- |
| LuxR1 int –Pkn-F | aggtaccTCCAATGTCGACGA | This work |
| LuxR1 int-Pkn-R | actcgagCACTTCAAGTTCAC | This work |
| LuxR2 int –Pkn-F | aggtaccGTCGTTGCTGTCAT | This work |
| LuxR2 int-Pkn-R | actcgagCGCATCTTCTGGTC | This work |
| Pr EXR3 act Kpn | aggtaccAGGGGTTGCCTGATTG | This work |
| Pr EXR3 act Hn1 | aaagcttCTCTTGTCGGCAATGCTTGAG | This work |
| Pr EXR3 act Hn2 | aaagcttTGGCAGACGGACAACCCG | This work |
| Pr EXR3 act Xba | atctagaTAATGGCTACGTGCTGCGC | This work |
| pBR R1act Kpn | aggtaccGCAGTGCTCAATTCTGG | This work |
| pBR R1act Xba | atctagaTGCTGCAAAGCCAGCACC | This work |
| pBR R2act Kpn | aggtaccGATGCTGTGACAGAAGTTTC | This work |
| pBR R2act Xba | atctagaTCGTCATGTCTGCCATGTCG | This work |
| pBR R3act Kpn | aggtaccGCAACGAACATGAGCGG | This work |
| pBR R3act Xba | atctagaATCACCCGGTCGCGCAG | This work |
| pr R1-Bam | aggatccTTGGTTGCGCTTTCTG | This work |
| pr R1-Xho | actcgagGCCATGAGAATGTAGCC | This work |
| pr R2-Bam | aggatccAGCGTAGATGCCG | This work |
| pr R2-Xho | actcgagAAGTTTCAGCAGAACCG | This work |
| pr R3-Bam | aggatccGTAGACGGTGCAGTGC | This work |
| pr R3-Xho | actcgagGGCGTGCAGTTCCAC | This work |
| pr Pip Bam | aggatccTTGATCGTCATGTC | This work |
| pr Pip Xho | actcgagCCGTTCACTACCGT | This work |
