# Supplementary material for: The Kiwifruit Emerging Pathogen Pseudomonas syringae pv. actinidiae Does Not Produce AHLs but Possesses Three LuxR Solos
Source: PLoS One. 2014 Jan 31;9(1):e87862. doi: 10.1371/journal.pone.0087862 (PMC3909224; doi:10.1371/journal.pone.0087862)
Supplement: Table S3 — Flanking genes to psaR1, psaR2 and psaR3 in Pseudomonas syringae pv. Actinidiae. (DOC) [file pone.0087862.s004.doc]

***Table S3. Flanking genes to psaR1, psaR2 and psaR3 in Pseudomonas syringae pv. a***ctinidiae

| ***luxR* solos** | **LuxR solo and flanking protein products** | **Nucleotide sequence length** |
| --- | --- | --- |
| ***psaR1*** | Hypothetical protein" | complement (19172..19690) |
|  | Ankyrin domain-containing protein" | complement (19793..20338) |
|  | Hypothetical protein" | 20700..20978 |
|  | **LuxR family transcriptional regulator"** | **complement (21129..21851)** |
|  | HAD-superfamily hydrolase" | complement (22126..22809) |
|  | ARD/ARD' family protein" | complement (22826..23371) |
|  | Methylthioribulose-1-phosphate dehydratase" | complement (23453..24067) |
|  | Transporter" | complement (24064..25248) |
|  | Chorismate synthase" | complement (25265..26356) |
|  | Hypothetical protein" | 26498..27529 |
| ***psaR2*** | Methyl-accepting chemotaxis protein" | complement (1..1305) |
|  | Sodium-proton antiporter" | complement (1542..2720) |
|  | Peptide-binding protein" | complement (2879..4393) |
|  | ATP-binding protein of peptide ABC transporter" | complement (4443..5423) |
|  | ATP-binding protein of peptide ABC transporter" | complement (5420..6439) |
|  | Permease of Peptide ABC transporter" | complement (6436..7299) |
|  | Permease of Peptide ABC transporter" | complement (7296..8243) |
|  | **Transcriptional regulator of LuxR family"** | **8582..9346** |
|  | Proline iminopeptidase"**#** | 9441..10382 |
|  | Methyl-accepting chemotaxis protein" | 10640..12265 |
|  | GAF domain protein" | 12510..13010 |
|  | GAF domain/GGDEF domain/EAL domain protein" | 13374..15197 |
|  | Membrane protein" | 15274..16074 |
|  | Conserved hypothetical protein" | 16299..16499 |
|  | Conserved domain protein" | complement (16745..16939) |
| ***psaR3*** | Hypothetical protein | complement (871..1488) |
|  | Putative transcriptional activator | complement (1492..2175) |
|  | GCN5-related N-acetyltransferase | complement (2177..2722) |
|  | Putative anthranilate synthase component I | 4717..6183 |
|  | Anthranilate synthase component II | 6180..6773 |
|  | **Putative LuxR family regulatory protein** | **6831..7673** |
|  | Phage integrase family site specific | 7770..7925 |

Bold letters indicate the respective LuxR solos of *Psa*

**^#^** Proline iminopeptidase is known to present neighboring with PAB solo
